# Effect of magnesium oxide on mechanical behavior of jute fiber reinforced epoxy bio nanocomposite

**DOI:** 10.1038/s41598-025-34078-0

**Published:** 2026-01-13

**Authors:** Md. Abdullah, Md Mahadi Hassan Parvez, Hassan Parvez, Robiul Hossen, Sree. Sourov Kumar, Nur Ahmed Tuhin, Abu Daud Anam

**Affiliations:** Department of Mechanical Engineering, City University, Khagan, Savar, Dhaka, 1340 Bangladesh

**Keywords:** Jute fiber, Epoxy composite, Magnesium oxide (MgO) nanoparticles, Bio nanocomposites, Mechanical properties, Engineering, Materials science

## Abstract

This work investigates how magnesium oxide (MgO) nanoparticles affect the mechanical performance of jute fiber–reinforced epoxy bio-nanocomposites. Three composite variations were generated using hand lay-up (HLU): one without MgO, one with 2% MgO, and another with 4%. The laminate surface was uniformly subjected to a controlled load of 30 kg using a flat steel plate and mechanical weights to ensure homogeneous bonding. All samples were cured accordingly. Tensile and flexural tests as well as Fourier Transform Infrared Spectroscopy (FTIR) and Scanning Electron Microscope (SEM) were used together to evaluate structural and chemical properties. Tensile test results revealed that the MgO-free composite exhibited higher tensile strength and stiffness, whereas the 2% MgO composite showed higher strain at failure, indicating improved ductility and toughness. This highlights a clear trade-off between strength and ductility depending on MgO content. Though all samples showed similar flexural strength, which is 39 MPa, flexural analysis revealed that the MgO-free composite had the greatest flexural modulus, which is 320.542 MPa, while the 2% MgO composite showed the lowest, which is 264.123 MPa. FTIR verified interactions between MgO and the epoxy matrix on a chemical level. Peaks at 3286 cm⁻¹, 1712 cm⁻¹, and 1264 cm⁻¹ in the MgO-free composite matched hydroxyl, carbonyl, and ester groups. These slightly changed peaks in the 2% MgO composite point to improved bonding. Reduced dispersion efficiency and further peaks at 350 cm⁻¹ were shown by the 4% MgO sample. These results were validated by SEM pictures displaying homogeneous MgO distribution at 2% and notable aggregation at 4%. Using without MgO was the best choice because it increased tensile strength and chemical bonding while keeping the structure strong, making it a good option for eco-friendly industrial applications.

## Introduction

 The increasing environmental concerns and the growing demand for sustainable materials have accelerated research into bio-based composites, particularly those reinforced with natural fibers. Among various natural fibers, jute has gained significant attention due to its low cost, biodegradability, good mechanical properties, and widespread availability^1^. When embedded in polymer matrices such as epoxy resins, jute fibers contribute to the development of eco-friendly composites suitable for a range of structural and semi-structural applications. However, natural fiber-reinforced polymer composites often exhibit limitations such as poor interfacial bonding between the hydrophilic fibers and the hydrophobic matrix, as well as relatively lower mechanical properties compared to synthetic fiber composites^2,3^. To address these challenges, the incorporation of nanomaterials has emerged as an effective strategy to enhance the interfacial adhesion and overall mechanical performance of bio-composites. Magnesium oxide (MgO) nanoparticles, due to their unique physicochemical properties, have shown promise as reinforcing agents in polymer nanocomposites^4^. Their high surface area, thermal stability, and mechanical strength make them suitable for improving the stiffness, tensile strength, and impact resistance of composite materials. Additionally, MgO possesses inherent biocompatibility and antimicrobial properties, which may further expand the functional applications of the developed composites^5^.

The magnesium oxide (MgO) in jute fiber composites, particularly in polypropylene matrices, has been shown to enhance fire retardancy properties without significantly compromising mechanical performance. In a study on jute fiber reinforced polypropylene composites, the addition of MgO improved fire retardancy metrics such as ignition time and total firing time, with 30% MgO content yielding an ignition time of 8 s and a total firing time of 268 s^6,10^. While the primary focus of this study was on fire retardancy, the mechanical properties such as tensile strength and modulus were also evaluated, indicating that the inclusion of MgO did not adversely affect these properties^7^. This aligns with findings from other studies that emphasize the importance of fiber treatment in enhancing the mechanical properties of jute fiber composites. For instance, alkali treatment of jute fibers has been shown to improve tensile strength and adhesion with matrices, whether in cement or epoxy, by removing hemicellulose and enhancing surface morphology^6,7^. These treatments are crucial for improving the mechanical behavior of jute fibers in composite applications, as untreated fibers often suffer from poor bonding and fiber pull-out^6,8^. Additionally, the durability of jute fibers in alkaline environments, such as those found in cementitious composites, is a concern, and ongoing research is assessing their long-term performance^9^. Overall, while magnesium oxide enhances fire retardancy, the mechanical behavior of jute fiber composites can be significantly improved through appropriate fiber treatments, ensuring better performance in various applications^6,7,10^.

The addition of magnesium oxide (MgO) to jute fiber-reinforced epoxy bio-nanocomposites significantly enhances their tensile strength, as evidenced by various studies on similar composite systems. The incorporation of MgO nanoparticles into epoxy matrices generally improves mechanical properties due to better stress transfer at the fiber-matrix interface. For instance, the addition of MgO to epoxy composites has been shown to increase tensile strength, with optimal results observed at specific weight fractions, such as 25 wt% in an epoxy-MgO composite, which achieved a tensile strength of 0.48 N/mm²^11^. Similarly, the inclusion of nano MgO fillers in banana fiber-reinforced epoxy composites resulted in enhanced tensile properties, attributed to improved dispersion and interfacial bonding^12^. In the context of jute fiber composites, the use of nanofillers like MgO, combined with surface treatments, has been reported to significantly improve mechanical properties, including compressive and interlaminar shear strengths, indicating a potential increase in tensile strength as well^13,14^. Furthermore, functionalized MgO nanoparticles have been shown to enhance the mechanical properties of epoxy composites by forming strong interfacial bonds, which likely contributes to increased tensile strength in jute fiber-reinforced systems as well^15^. Overall, the integration of MgO into jute fiber-reinforced epoxy composites is expected to improve tensile strength through enhanced interfacial interactions and stress distribution.

The optimal concentration of Magnesium Oxide (MgO) for enhancing the impact resistance of Jute Fiber–Reinforced Epoxy Bio-Nanocomposites appears to be around 3% based on findings from various studies. Research indicates that this concentration significantly improves mechanical properties, including compressive and interlaminar shear strengths, while also reducing water absorption in alkaline-treated and untreated jute fiber composites^16,17^. Additionally, studies on MgO nanosheets suggest that a concentration of 0.2 wt% optimally enhances fracture toughness, indicating that lower concentrations may also be effective in improving impact resistance^18^. Furthermore, other studies have shown that higher concentrations, such as 8%, can lead to increased impact strength, although the relationship may become nonlinear^19^. Thus, while 3% is optimal for certain properties, the specific application and desired mechanical characteristics may dictate the best concentration of MgO in jute fiber composites^17,19,20^.

The incorporation of Magnesium Oxide (MgO) significantly enhances the thermal stability of Jute Fiber–Reinforced Epoxy Bio-Nanocomposites through several mechanisms. MgO acts as a flame retardant, improving the thermal stability of epoxy composites by reducing flammability and enhancing heat resistance, as evidenced by studies showing that metal oxide additives, including MgO, improve thermal properties when used in appropriate percentages^21^. Furthermore, functionalized MgO nanoparticles have been shown to create strong interfacial bonds with the epoxy matrix, leading to improved mechanical and thermal properties, including a tenfold increase in thermal conductivity compared to neat epoxy^22^. Additionally, the treatment of Jute fibers with Mg^+ 2^ enhances their flame retardancy and interfacial bonding with the matrix, further contributing to the overall thermal stability of the composite^23^. Overall, the synergistic effects of MgO and treated Jute fibers result in a composite with superior thermal performance, making it suitable for various applications^24,25^.

Magnesium oxide (MgO) into jute fiber-reinforced epoxy bio-nanocomposites offers several advantages and disadvantages, as evidenced by studies on similar composite systems. The primary advantage of adding MgO is the enhancement of mechanical properties. For instance, MgO addition has been shown to increase tensile strength, impact resistance, and hardness in epoxy composites, with optimal results observed at specific weight ratios, such as 25 wt% in some studies, which yielded the highest tensile strength and impact resistance values^1,26^. Furthermore, functionalized MgO nanoparticles improve the interfacial bonding between the MgO and epoxy matrix, leading to enhanced tensile strength, modulus of elasticity, and ductility, as well as significantly improved thermal conductivity^2,27,28^. Additionally, the use of two-dimensional MgO nanosheets has been found to enhance fracture toughness, with a 47% improvement at 0.2 wt% MgO, indicating better resistance to crack propagation^3,28,29^. However, there are potential disadvantages, such as the need for precise control over MgO content and dispersion to avoid agglomeration, which can negatively impact the composite’s mechanical properties. Moreover, while MgO enhances certain properties, the overall performance is highly dependent on the specific composite formulation and processing techniques used^5,30^. Therefore, while MgO incorporation can significantly improve the mechanical behavior of jute fiber-reinforced epoxy bio-nanocomposites, careful consideration of the composite design and processing is crucial to fully realize these benefits.

The primary environmental challenges associated with the use of magnesium oxide in jute fiber–reinforced epoxy bio-nano composites include degradation due to moisture absorption, temperature fluctuations, and exposure to corrosive environments. Jute fibers, while offering sustainability benefits, are susceptible to moisture, which can lead to reduced mechanical properties and structural integrity over time^31,32^. Additionally, magnesium oxide nanoparticles, although enhancing the mechanical and antibacterial properties of composites, face challenges related to their high degradation rates in biological environments, which can compromise the longevity of the composite materials^32^. Furthermore, the interaction between jute fibers and the epoxy matrix can be adversely affected by environmental factors, leading to issues such as fiber-matrix adhesion loss and overall composite performance degradation^33,34^. Thus, addressing these environmental challenges is crucial for the effective application of magnesium oxide in jute fiber composites.

The incorporation of Magnesium Oxide (MgO) into jute fiber reinforced epoxy bio-nanocomposites significantly enhances their thermal stability and degradation resistance. The presence of MgO, as demonstrated in various studies, contributes to improved thermal properties by forming a protective char layer that restricts oxygen access, thereby enhancing flame retardancy and reducing thermal degradation^35^. This is further supported by the findings that MgO, when used as a filler in composites, can significantly increase thermal conductivity due to its ability to form highly ordered structures, which facilitate heat dissipation^36^. Additionally, the integration of jute nanofibers within the epoxy matrix has been shown to improve thermal stability, as these fibers provide resistance to thermal degradation and enhance the mechanical properties of the composite^37,39^. The dynamic mechanical analysis of these composites reveals an increase in storage modulus and a decrease in mechanical loss factor, indicating enhanced interfacial adhesion and compatibility between the nanofibers and the matrix, which contributes to the overall thermal stability^38^. Furthermore, the use of magnesium compounds, such as magnesium carbonate hydroxide pentahydrate, in similar composite systems has been shown to improve both mechanical and thermal properties, suggesting that MgO could have a similar effect in jute fiber reinforced epoxy composites^39,40^. Overall, the incorporation of MgO into these composites not only enhances thermal stability but also contributes to improved mechanical robustness and flame resistance, making them suitable for high-performance applications.

The incorporation of Magnesium Oxide (MgO) into Jute Fiber Reinforced Epoxy Bio-Nanocomposites can significantly influence their biodegradability and recyclability. MgO has been shown to enhance the mechanical properties of composites, which is crucial for structural applications, while also potentially improving the interfacial bonding between jute fibers and the epoxy matrix^41^. This enhancement can lead to better performance in terms of durability and strength, which is essential for practical applications^42^. Furthermore, the presence of MgO may facilitate the chemical degradation processes, thereby promoting the biodegradability of the composite materials, as jute fibers are already known for their natural biodegradability^43,44^. The combination of MgO with jute fibers could also support recycling efforts, as treated jute fibers have demonstrated potential for re-impregnation and reuse in new composite formulations^45,47–52^. Overall, the integration of MgO could optimize both the environmental sustainability and lifecycle management of jute fiber reinforced epoxy composites.

The novelty of this work lies in the systematic development and characterization of jute fiber–reinforced epoxy bio-nanocomposites incorporating low weight fractions of magnesium oxide (MgO) nanoparticles (2 wt% and 4 wt%) using a simple and cost-effective hand lay-up (HLU) technique. Unlike many previous studies that focus either on fire retardancy, high filler loading, or different polymer matrices, this study emphasizes the combined effect of recycled jute fibers (sourced from waste jute bags) and nano-scale MgO on the mechanical, microstructural, and chemical properties of epoxy composites. The work uniquely explores the balance between sustainability, material performance, and processing simplicity by integrating waste-derived natural fibers with nanofillers at optimized concentrations, avoiding excessive filler agglomeration. Furthermore, the concurrent evaluation of tensile, flexural, SEM, and FTIR analyses provides a comprehensive understanding of fiber–matrix–nanofiller interactions, highlighting the role of MgO in enhancing interfacial bonding and structural integrity. This approach contributes new insights into the design of eco-friendly, mechanically improved bio-nanocomposites suitable for structural and semi-structural applications, while promoting waste utilization and sustainable material development.

This study aims to evaluate the effect of MgO nanoparticles on the mechanical behavior of jute fiber–reinforced epoxy bio-nanocomposites. The hybridization of natural fibers with inorganic nanoparticles is expected to bridge the gap between performance and sustainability, paving the way for the development of lightweight, durable, and environmentally friendly materials for engineering applications. This work contributes to the growing body of knowledge on bio-nanocomposites and their potential to address the challenges of conventional synthetic composites in structural and industrial applications.

## Materials and methodology

### Materials

**Fig. 1:**
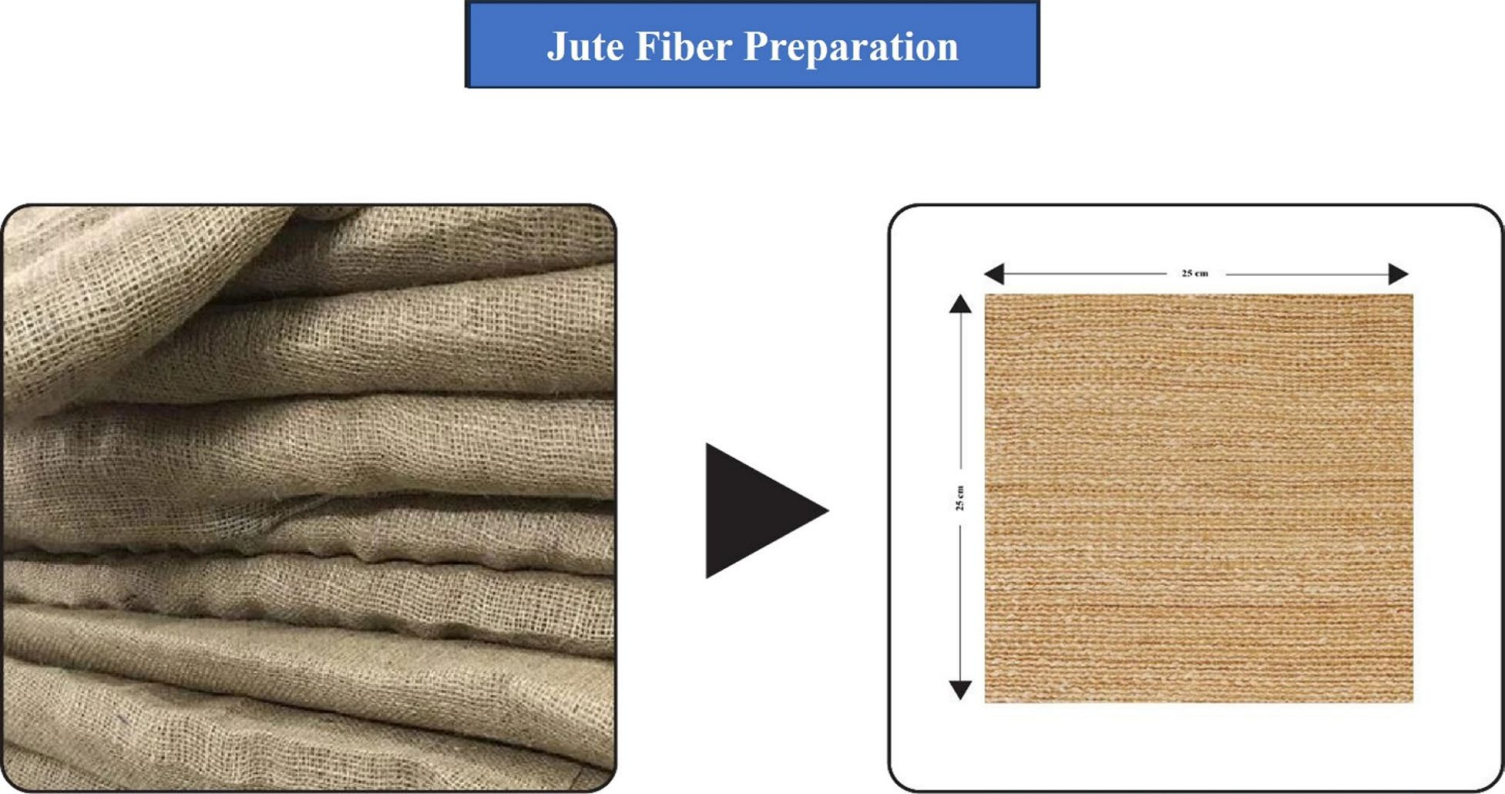
Preparation of jute fiber.

For the synthesis of the composite, there are used Epoxy resin, Epoxy hardener, Jute fiber, and MgO. Jute is collected from used and spoilt jute bags, washed properly, dried in the sun, and cut into desired sizes by using scissors. Figure 1 shows the preparation of jute fiber. MgO (nanomaterial), Epoxy resin, and Epoxy hardener are purchased from the local market which was supplied by Modern Scientific Ltd. Rasel Center, 27 Hatkhola Rd, Dhaka 1203, Bangladesh.

### Process of jute fiber composite synthesis by HLU process

**Fig. 2:**
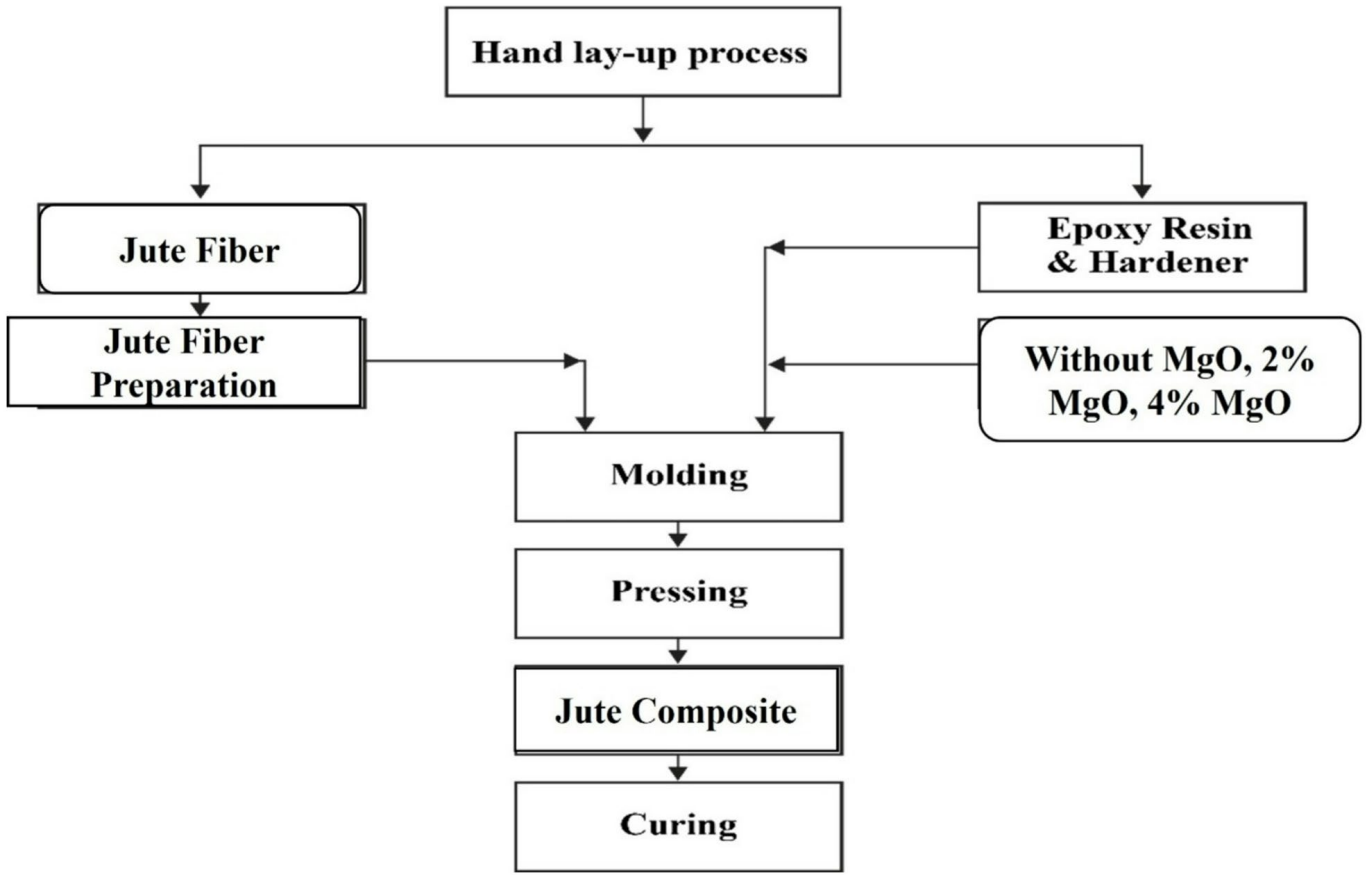
Block diagram of jute composite with 2% MgO filler, 4% MgO filler and without MgO.

**Fig. 3:**
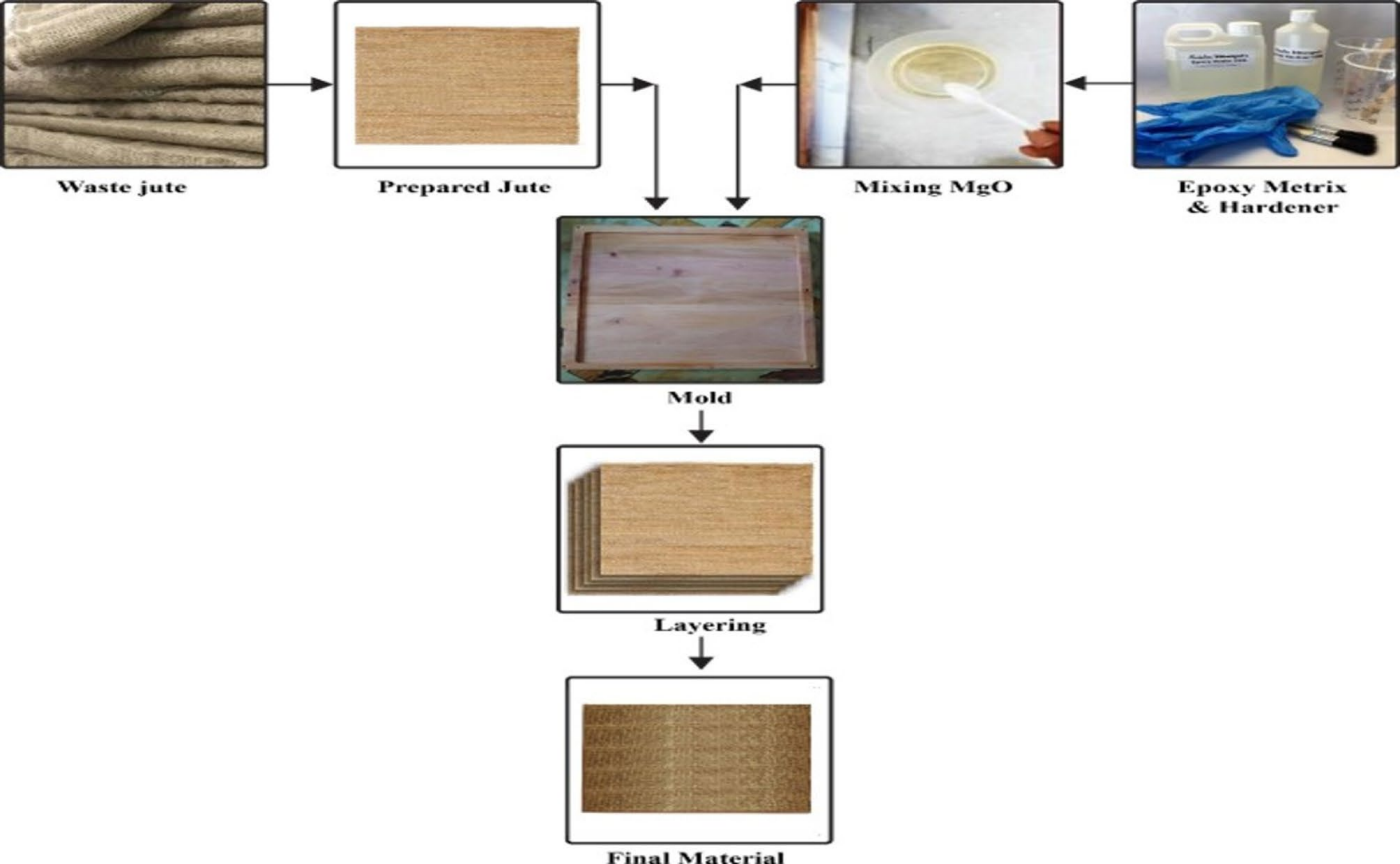
Synthesis process of Jute composite by HLU process.

Table 1 outlines the components used to synthesize three distinct types of composites. All materials were carefully and accurately measured using an electronic balance at the start of the process. The measured components were then thoroughly mixed and blended, as depicted in Fig. 2. The hand lay-up (HLU) technique, illustrated in Fig. 3, involved brushing the prepared epoxy resin mixture onto the mold surface prior to placing the jute fiber layer to ensure optimal impregnation. The jute layers, each cut to fit a 300 mm × 300 mm mold, were then placed sequentially, with each layer followed by the application of additional epoxy resin. This layer-by-layer impregnation continued until the desired laminate structure was achieved. The laminated structure was subsequently rolled to eliminate trapped air bubbles and ensure uniform resin distribution. The composite was then left to cure at room temperature for 72 h under a constant load of 30 kg to promote proper gelation and improve compression of the unsaturated polyester (UPE) matrix. Table 2 provides the chemical composition of jute fiber^53^.

**Table 1:** Material used for the synthesis of composite.

Sample Name	Epoxy Resin and Hardener (wt%)	Epoxy Resin-Hardener Ratio	Jute fiber (wt%)	MgO (wt%)
C 1	70	10: 1	30	0
C 2	70	10: 1	28	2
C 3	70	10: 1	26	4

**Table 2:** A typical chemical composition of jute fiber^53^.

Component	Typical Content (% by weight)
Cellulose	58–63
Hemicellulose	20–24
Lignin	12–13
Pectin	1–2
Waxes and Fatty substances	1–2
Ash	0.5–1

### Characterization

#### Tensile test

Tensile testing provides critical information about the tensile strength, yield strength, and ductility of composite materials. It measures the force required to break the composite and the extent to which the specimen stretches or elongates before failure. Typically, tensile testing of composites is conducted using basic tension or flat-sandwich tension methods in accordance with ASTM D3039 − 14 standards. Standard specimen dimensions for such tests are 250 mm × 15 mm. The resulting stress-strain diagrams are used to determine the tensile modulus of the material. Tensile strength is defined as the maximum stress sustained by the composite before fracture. Stiffness is represented by Young’s modulus, obtained from the linear elastic region of the stress–strain curve. Ductility refers to the ability of the material to undergo plastic deformation and is measured by strain at fracture.

#### Flexural test

Flexural testing was conducted using a universal testing machine (Model STM-50, Santam, Tehran, Iran). The distance between the support spans was set at 40 mm, and a loading force was applied at a crosshead speed of 5 mm/min until the specimens fractured. Both the loading and supporting plungers had a diameter of 20 mm. Flexural strength, expressed in megapascals (MPa), was calculated based on the maximum load recorded during testing using the following formula:

$$Flexural\, Strength, F= \frac{\mathrm{3WL}}{\mathrm{2bd}^2}$$

Here,

W = load at fracture; L = distance between supporting points (40 mm); b = width of specimens (mm); d = specimen thickness (mm).

#### SEM test

A Hitachi S-4800 scanning electron microscope was used to examine the microstructure of the bioplastic composites. Two distinct samples were cryo-fractured after immersion in liquid nitrogen and subsequently broken at random to expose their internal surfaces. The fractured specimens were mounted on aluminum stubs and secured using double-sided adhesive tape. Prior to imaging, the samples were sputter-coated with a thin layer of gold-palladium to enhance conductivity. Microstructural analysis was carried out at an accelerating voltage of 10 kV with a working distance of 10 mm.

#### FTIR test

Fourier transform infrared spectroscopy (FTIR) was employed to identify the chemical functional groups present in the composites. The analysis was performed using a PerkinElmer FT-IR spectrometer. The PerkinElmer FTIR spectrometer is utilized for testing. The absorbance range examined in this study extended from 4000 to 350 cm^− 1^. Table 3 shows the FTIR analysis data table of synthesized bio composite.

**Table 3:** FTIR analysis data table of synthesized bio composite.

Peak No.	Wavenumber (cm⁻¹)	Assignment	Functional Group/Vibration
1	4000–3300	Hydrogen bonded hydroxyl/amino groups	O–H/N–H Stretching
2	2920–2850	Methylene group in polymer backbone	C–H Stretching (aliphatic)
3	1740–1720	Ester or carboxylic acid groups	C = O Stretching
4	1650–1620	Unsaturated carbon or protein-related amide	C = C or Amide I (C = O Stretching)
5	1550–1530	Proteinaceous material or peptide linkage	N–H Bending/Amide II
6	1450–1410	Aliphatic hydrocarbon groups	CH₂/CH₃ Bending
7	1250–1020	Ether linkages/polysaccharide or ester bonds	C–O–C/C–N Stretching
8	900–600	Aromatic components/polysaccharide fingerprint	C–H Bending/Aromatic ring vibrations
9	512 − 350	Halo compound	C-l/Strong stretching

## Results and discussion

### FTIR (Fourier transformed infrared spectroscopy) analysis

**Fig. 4:**
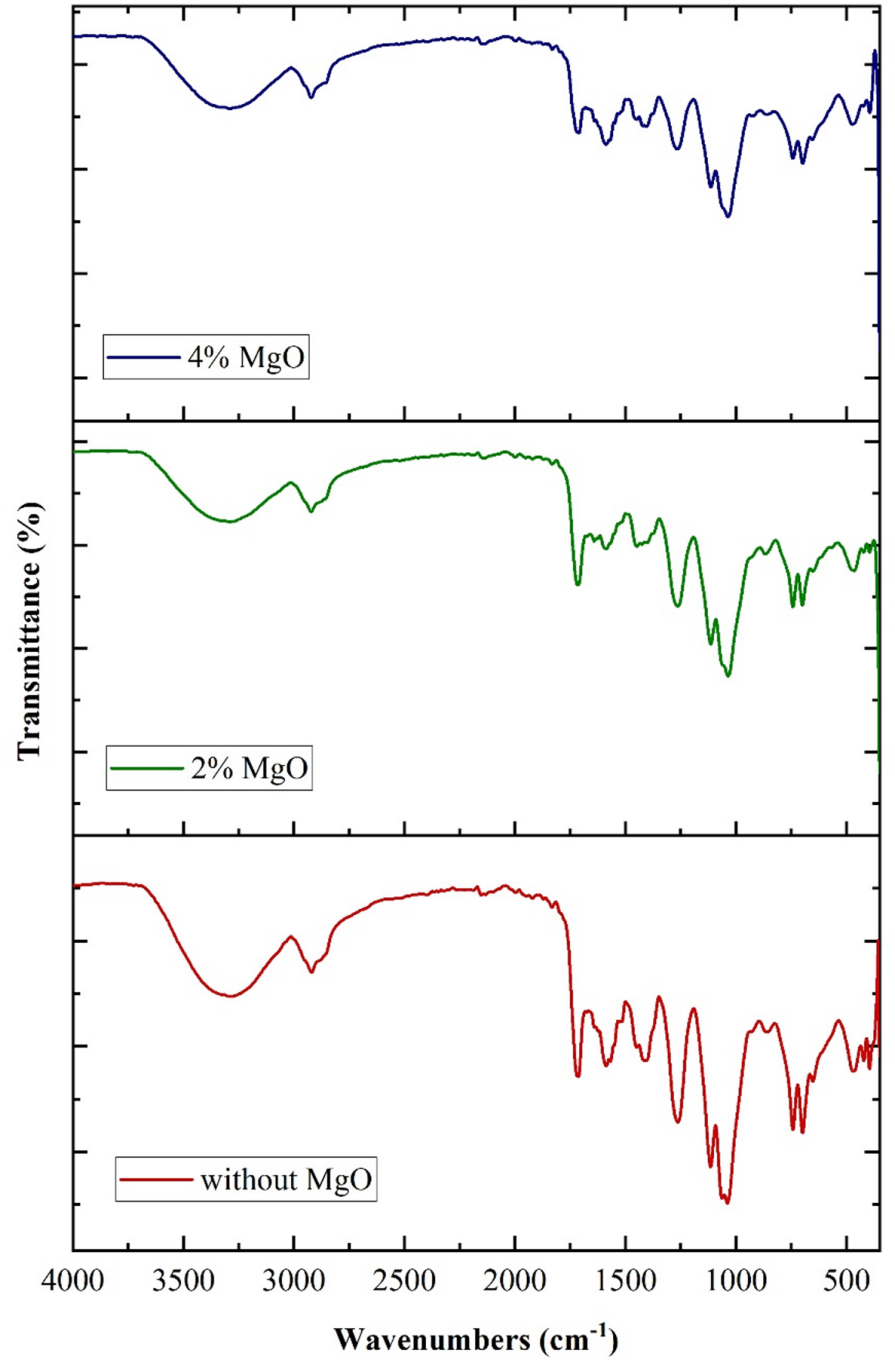
FTIR of Jute composite.

The FTIR spectra in the provided graph illustrate the comparative transmittance profiles of three fabricated jute fiber composites; without MgO, with 2% MgO, and with 4% MgO; across a wavenumber range of 4000 to 500 cm^− 1^, as shown in Fig. 4. These spectra show how the addition of MgO nanoparticles influences the structural properties and chemical bonding of the jute fiber composites. The O–H stretching vibrations of hydroxyl groups are represented by the distinctive broad absorption bands that are seen in all samples at about 3300–3400 cm^− 1^. As the MgO content increases, these bands become less intense, indicating that the hydroxyl groups in the jute matrix and MgO nanoparticles are interacting. Sharper peaks in the 1000–500 cm^− 1^ range are particularly notable in the 2% MgO composite, which could be a sign of improved matrix interaction and Mg–O stretching vibrations. The 4% MgO composite exhibits additional increasing and shift in this area, but the spectrum as a whole is flatter, indicating its less consolidated structure, which is in line with its reported excessive softness and unsuitability for tensile testing. The FTIR analysis shows structural changes and validates the successful incorporation of MgO. While 4% MgO results in over-saturation and reduced material integrity, 2% MgO exhibits optimal interaction. The decrease in intensity of O–H stretching band between 3300 and 3400 cm^− 1^ and negligible shifts in C–O–C and C = O stretching bands confirm that MgO nanoparticles are actively involved in bonding with the epoxy matrix at the interface. Decreased intensity of O–H indicates that hydroxyls of jute fiber have reacted with surface hydroxyl or oxygen vacancies of MgO to form new Mg–O–C linkages for chemical adhesion. In addition, enhanced sharpness and resolution of peaks between 1000 and 500 cm^− 1^ again verify the development of Mg–O and Mg–O–C vibrations, indicative of successful interfacial modification instead of physical mixing. The collective spectral changes all verify that the 2% MgO composite had better chemical compatibility and crosslinking density of the fiber–matrix interface, in accordance with its increased ductility and toughness as demonstrated in mechanical tests.

### SEM (scanning electron microscopy)

**Fig. 5:**
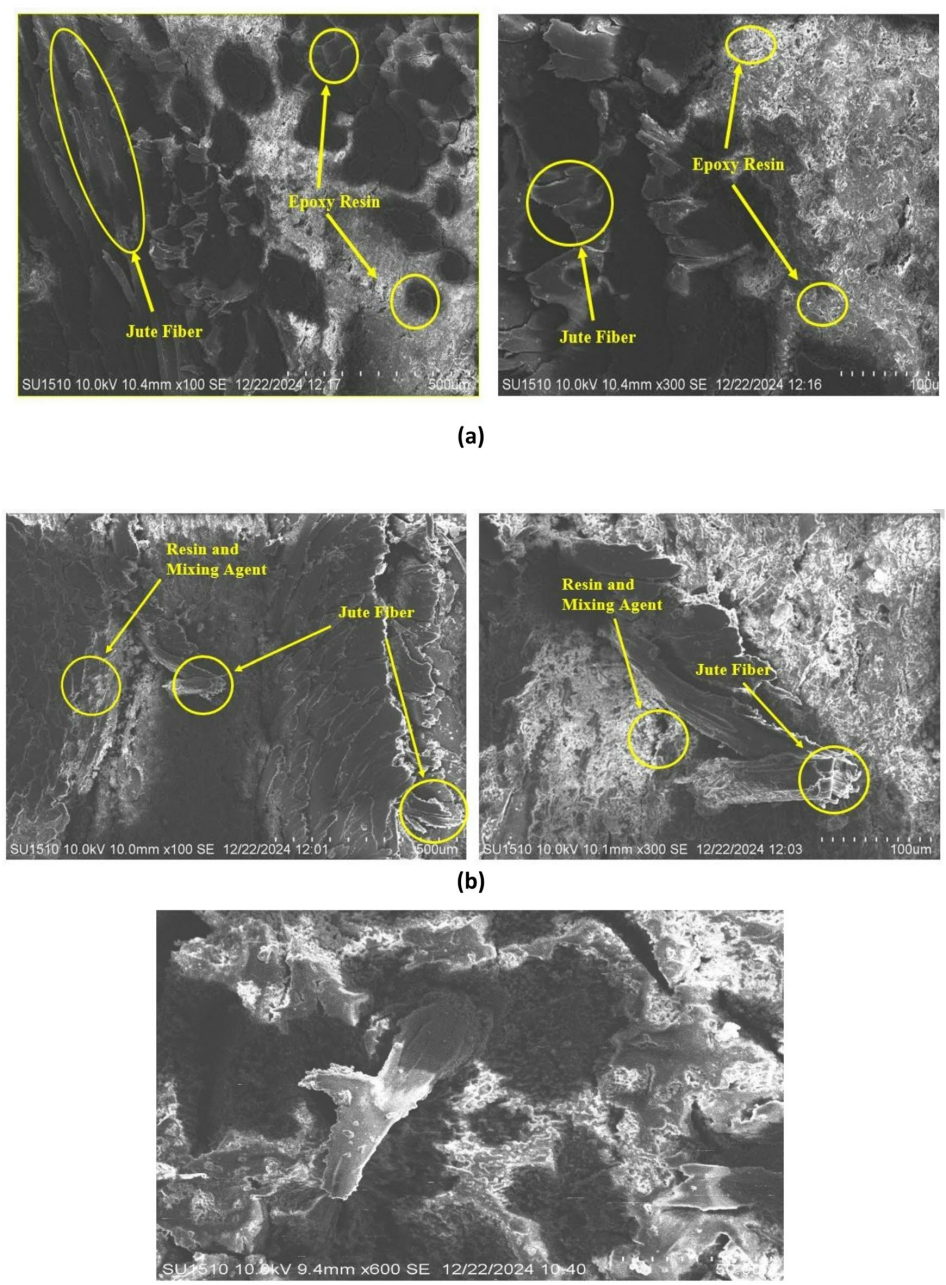

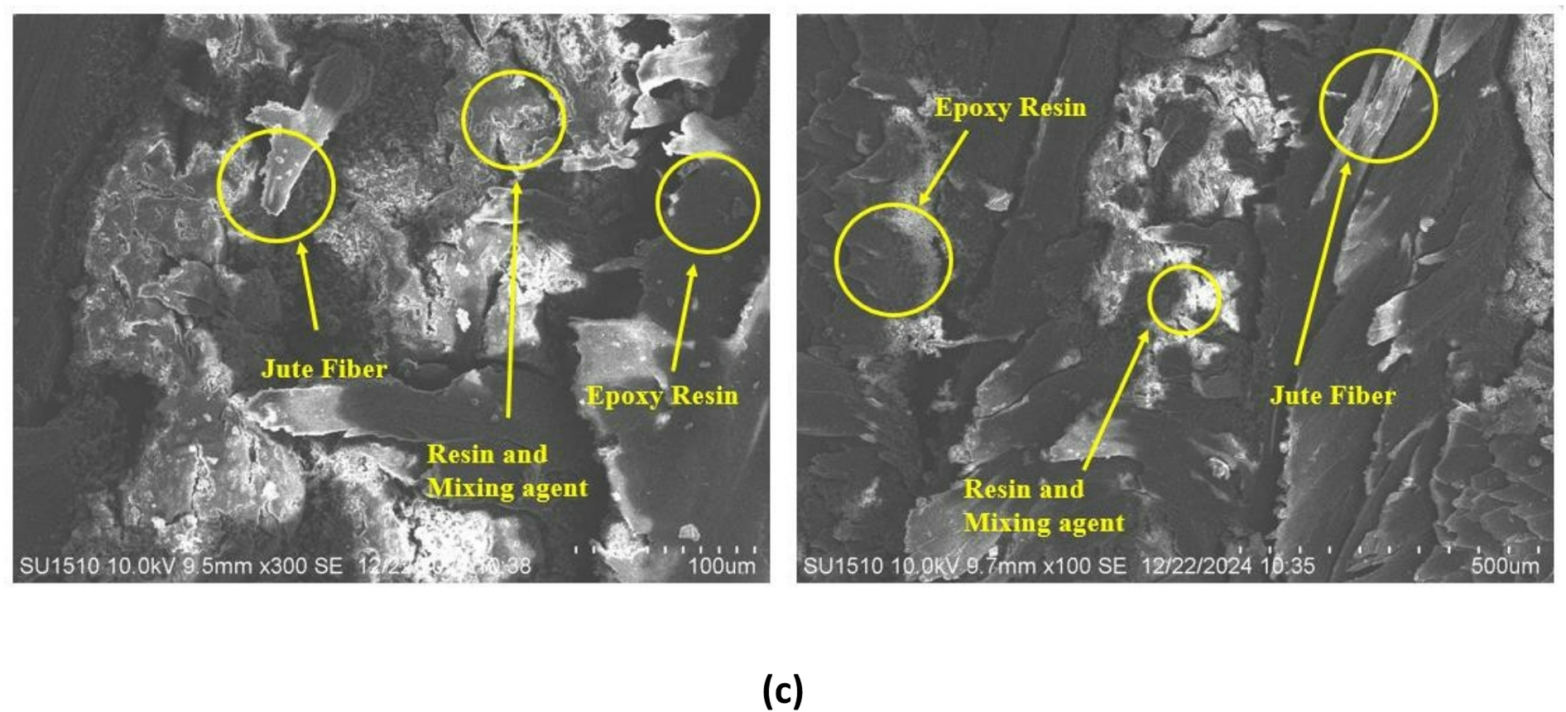
SEM of Jute composite (**a**) without MgO, (**b**) 2% MgO, (**c**) 4% MgO.

The surface morphology of jute-epoxy composites with different MgO content (0%, 2%, and 4%) at a magnification of 300x was evaluated using Scanning Electron Microscopy (SEM), shown in Fig. 5. The comparative study shows variations in microstructure, fiber distribution, and void formation resulting from the inclusion of MgO nanoparticles.

The jute composite without MgO’s shows relatively smooth surface. The jute fibers were well-embedded inside the epoxy matrix, and the absence of notable porosity indicated a homogenous structure with good fiber-matrix adhesion. Minor surface cracks, however, indicated variations during composite manufacture that could affect the thermal and mechanical performance. The SEM image showed a clearer and finer surface structure upon adding 2% MgO. Surface features were better seen with 100 nm pixel size. There were clear minor voids and fluctuations that seemed to be uniformly distributed. This suggests that MgO particles were evenly distributed all over the matrix. Though the general surface stayed smooth, implying better interfacial bonding and possible improvement in mechanical integrity, the interaction between MgO nanoparticles and the jute fibers could have caused these voids. The 4% MgO composite had a more irregular and porous surface. At higher magnifications, the jute fibers appeared globular with noticeable surface impurities. The natural structure of jute, which contains non-cellulosic materials including pectin’s, could cause these globules^46^. Increased MgO loading probably caused agglomeration, which lowered particle dispersion effectiveness.

Although 2% MgO increases dispersion and microstructure, 4% MgO adds surface contaminants and porosity that could influence composite performance.

### Tensile test

**Fig. 6:**
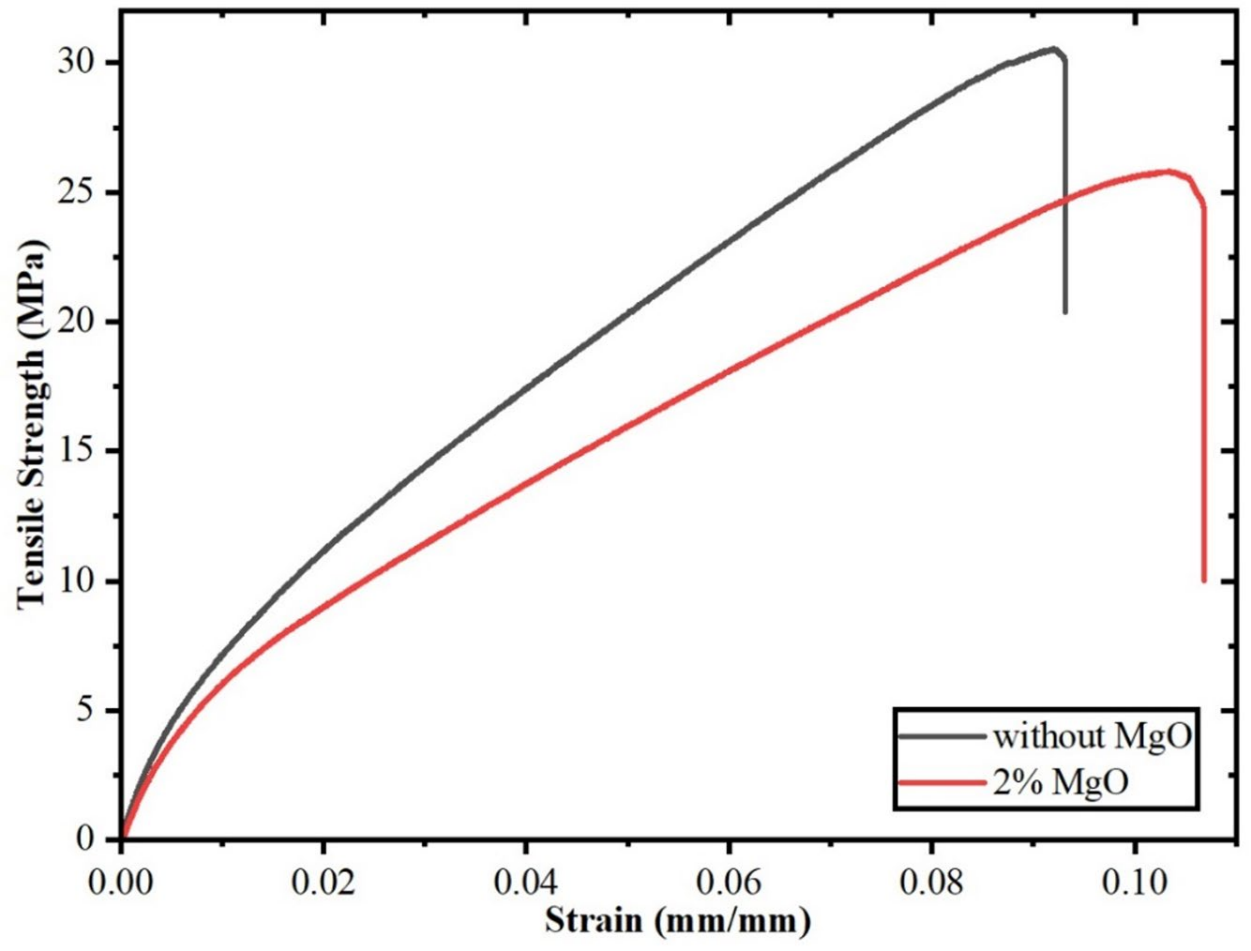
Stress-strain diagram of the fabricated jute fiber composite materials.

Figure 6 compares the mechanical properties of two jute fiber composites using a tensile test graph. The composite with 2% MgO has a slightly lower ultimate tensile strength of about 26.8 MPa, while the composite without MgO has a higher strength of about 29.4 MPa. This implies that the unreinforced composite is more brittle even though it can sustain a greater peak load before failures. The 2% MgO composite, on the other hand, shows significantly greater strain at fracture, suggesting better ductility and energy absorption capability. An essential property of ductile materials, the extended strain range for 2% MgO composite curve shows that the MgO-reinforced composite can undergo more plastic deformation before breaking. A sudden fracture, characteristic of brittle materials, is highlighted by the sharp drop in the black curve post-peak, whereas a more progressive failure mode is suggested by the red curve’s gradual decline. MgO nanoparticles considerably increase the material’s toughness and flexibility while slightly decreasing its tensile strength. This change in mechanical response suggests that MgO functions as a reinforcing agent, improving the fiber-matrix interaction and resulting in less brittle and more ductile behavior. The 2% MgO composite behaves like toughened nanocomposites, making it appropriate for applications needing ductility and resilience, while the unreinforced composite behaves like a traditional natural fiber composite with greater strength but a limited capacity for deformation. There were three samples were tested for each composite material to enhance statistical reliability. Tensile and flexural data variability were presented in mean values and standard deviation. Tensile strength results of 30.53 ± 0.77 MPa for the without MgO composite and 25.82 ± 1.04 MPa for the 2% MgO composite reveal equilibrated performance with minimal dispersion. The results indicate that MgO-reinforced specimens are ductile, although differences between the two composites were not statistically different (*p* > 0.05). Thus, it is clear that strength and ductility must be exchanged, without MgO increasing the jute fiber composite’s overall mechanical versatility. In this study, stiffness is evaluated using Young’s modulus, tensile strength refers to the maximum stress prior to fracture, and ductility is quantified by strain at failure. Although the MgO-free composite exhibits higher tensile strength and stiffness, the 2% MgO composite demonstrates superior ductility, indicating enhanced energy absorption capability. Therefore, neither composite dominates all mechanical parameters; instead, performance depends on application-specific requirements.

### Flexural test analysis

**Fig. 7:**
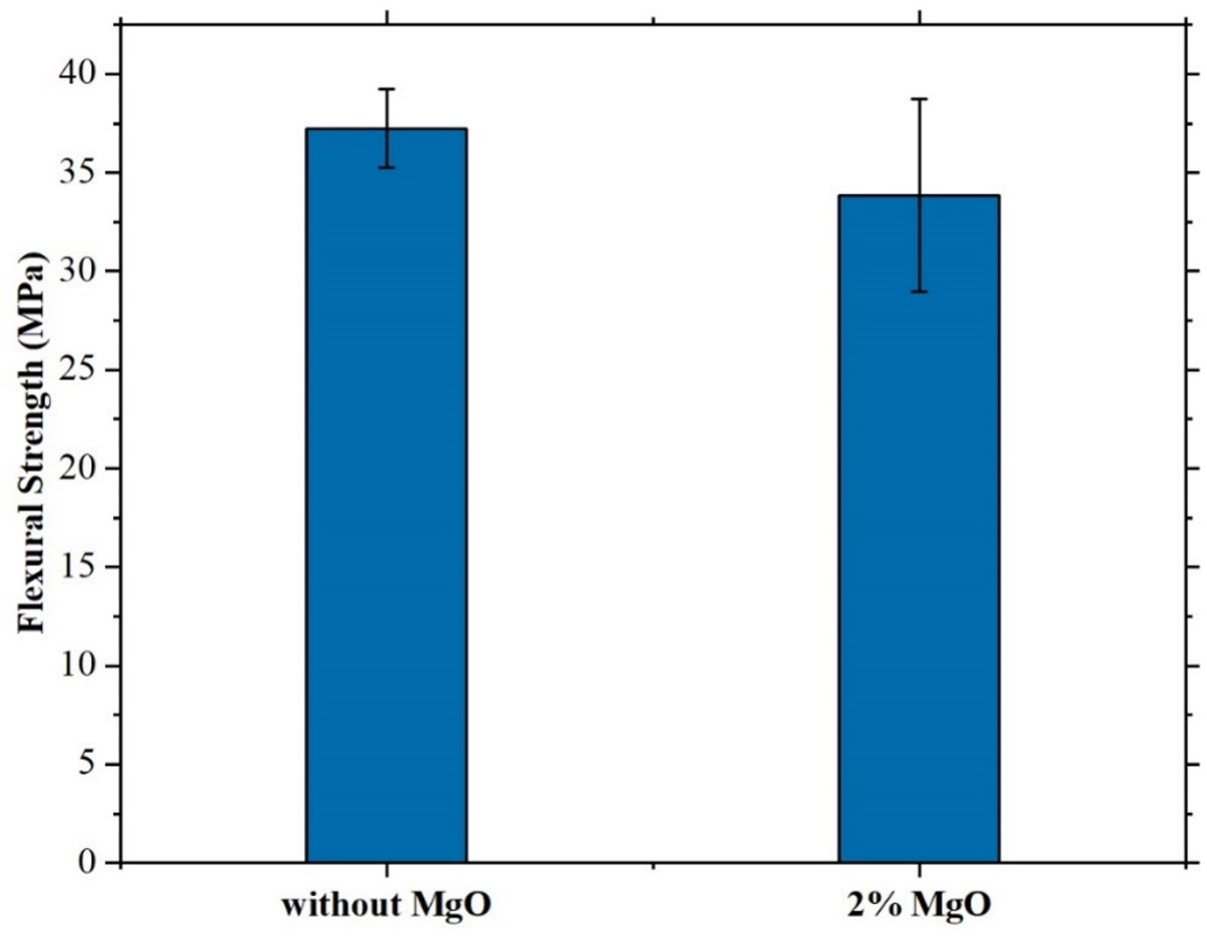
Flexural strength of the fabricated jute fiber composite materials.

The Fig. 7 shows the flexural strength of two jute fiber composites one with 2% MgO and the other without. With around 33.8 MPa, the composite with 2% MgO shows a little lower flexural strength than the one without MgO, which shows about 37.5 MPa. This suggests that the addition of MgO slightly lowers the bending stress resistance of the material. The error bars point to more variation in the 2% MgO composite, maybe caused by non-uniform distribution of MgO nanoparticles or matrix-fiber interface differences.

A consistent pattern develops when compared to the tensile test results. The composite without MgO showed greater tensile strength as well than the 2% MgO composite, suggesting that MgO inclusion slightly lowers both tensile and flexural strength. However, this reduction is not necessarily a disadvantage it comes with the benefit of improved ductility, as seen in the tensile stress-strain curve. The 2% MgO composite showed more strain at break, indicating improved energy absorption and deformation capacity, which is especially important in uses requiring toughness rather than peak strength. Though both tensile and flexural strength slightly drop with 2% MgO addition, the mechanical behavior changes toward a more ductile and tough material. Though stronger in both loading conditions, the composite without MgO is more brittle and less tolerant to deformation. When modifying materials to meet particular requirements in performance, this compromise between strength and ductility is typical^47^. The flexural results also include standard deviations to represent experimental variability, demonstrating that the observed reductions in strength fall within expected statistical limits (*p* > 0.05).

The Table 4 presents a comparative analysis of the fabricated jute fiber composites without MgO, with 2% MgO, and with 4% MgO based on FTIR, SEM, tensile, and flexural test results. It highlights the impact of MgO nanoparticle addition on the composites morphological, mechanical, and structural characteristics.

**Table 4:** Mechanical properties of the fabricated composites.

Tests/Properties	Without MgO	2% MgO	4% MgO
FTIR	■ Strong O–H peak ■ No Mg–O bond signals	■ Reduced O–H peak ■ Mg–O peaks evident ■ Better matrix bonding	■ Broad/flattened spectrum ■ Weak structure
SEM	■ Smooth surface ■ Good fiber-matrix adhesion ■ Minor cracks	■ Uniform MgO dispersion ■ Smooth surface with minor voids	■ Irregular surface ■ Fiber globules ■ Agglomeration, porosity
Tensile Strength (MPa)	30.53 ± 0.77	25.82 ± 1.04	Not tested (excessive softness)
Flexural Strength (MPa)	37.24 ± 2.0	33.86 ± 4.9	Not tested (excessive softness)
Material Behavior	Higher strength and stiffness but brittle	Slightly weaker but more ductile and tough	Soft, porous, and structurally unstable

## Conclusion

This study systematically investigated the influence of magnesium oxide (MgO) nanoparticles on the mechanical behavior and interfacial characteristics of jute fiber–reinforced epoxy bio-nanocomposites fabricated via a hand lay-up process. The results demonstrate that MgO content plays a decisive role in governing the balance between strength, ductility, and microstructural integrity of the composites.

The incorporation of a low MgO loading (2 wt%) altered the mechanical response of the composite from a relatively brittle behavior to a more ductile and toughened one. Although both tensile and flexural strengths showed a modest reduction compared to the MgO-free composite, the significant increase in strain at fracture and the more progressive failure mode indicate enhanced energy absorption capacity. This shift in behavior highlights that MgO functions more as a toughening and interfacial-modifying agent rather than a conventional strength-enhancing filler in jute–epoxy systems.

Microstructural and chemical analyses provided strong support for the mechanical trends. FTIR results revealed peak shifts and reduced hydroxyl intensity in MgO-containing composites, indicating improved physicochemical interactions between MgO, jute fibers, and the epoxy matrix. SEM observations confirmed that uniform nanoparticle dispersion and improved fiber–matrix adhesion were achieved at 2 wt% MgO, while excessive loading (4 wt%) led to particle agglomeration, increased porosity, and structural instability. The inability to mechanically test the 4 wt% MgO composites further emphasizes the detrimental effect of over-saturation with nanofillers.

An important outcome of this work is the identification of an optimal MgO loading threshold, beyond which composite performance deteriorates due to poor dispersion and weakened structural cohesion. The findings underline the necessity of controlled nanoparticle content and dispersion when designing natural fiber–based bio-nanocomposites.

Overall, this study demonstrates that incorporating 2 wt% MgO into jute fiber–reinforced epoxy composites effectively improve ductility, toughness, and interfacial bonding while maintaining acceptable strength levels. These characteristics make the developed composite particularly suitable for applications where resistance to deformation, impact tolerance, and sustainability are more critical than maximum load-bearing capacity. The use of recycled jute fibers and a simple fabrication process further strengthen the practical relevance of this work for developing environmentally responsible composite materials.

These enhancements suggest that low-percentage MgO inclusion optimizes the interfacial bonding between hydrophilic jute fibers and the hydrophobic epoxy matrix, addressing one of the longstanding challenges in natural fiber composite development while maintaining material sustainability.

In conclusion, the jute fiber–reinforced epoxy composite without MgO offers higher strength and stiffness, while the 2% MgO composite provides improved ductility and toughness. The use of waste-derived jute fibers, reduced nanoparticle loading, and enhanced mechanical durability collectively support the suitability of the 2% MgO composite for eco-friendly industrial applications such as lightweight panels, packaging, and automotive interior components.

### Industrial limitations and possible solutions

One of the primary industrial limitations of this work is the use of the hand lay-up (HLU) technique, which, although cost-effective and suitable for laboratory-scale fabrication, offers limited control over fiber alignment, resin distribution, and thickness uniformity. This can result in variability in mechanical properties between batches. To overcome this, industrial adoption would require transitioning to compression molding, resin transfer molding (RTM), or vacuum-assisted resin infusion (VARI), which provide better repeatability, improved fiber wetting, and reduced void content.

Another limitation is the dispersion sensitivity of MgO nanoparticles. As observed in the 4 wt% MgO composite, nanoparticle agglomeration leads to porosity, structural weakness, and loss of mechanical integrity. In an industrial setting, this dispersion challenge can be addressed through surface functionalization of MgO nanoparticles, high-shear mixing, or ultrasonication during resin preparation, ensuring uniform nanoparticle distribution even at slightly higher filler contents.

The moisture sensitivity of jute fibers poses another challenge for long-term industrial use, particularly in humid or outdoor environments. Moisture absorption can degrade fiber–matrix adhesion and reduce mechanical performance over time. This limitation can be mitigated by chemical surface treatments of jute fibers (alkali, silane, or acetylation treatments), or by applying protective surface coatings to finished composite parts to improve environmental durability.

Additionally, while epoxy offers excellent mechanical and chemical stability, it presents recyclability and end-of-life disposal challenges, which may limit large-scale adoption in sustainability-driven industries. Future industrial solutions could include replacing conventional epoxy with bio-based or recyclable epoxy systems and developing mechanical or chemical recycling routes for jute-based composites.

### Future scope of the work

The future scope of this work includes optimizing nanoparticle dispersion techniques and exploring functionalized MgO nanoparticles to further enhance interfacial bonding without compromising ductility. Investigating hybrid nanofillers (MgO combined with graphene, nanoclay, or silica) may allow simultaneous improvement of strength, toughness, and thermal resistance. Long-term aging, moisture absorption, and fatigue studies are essential to validate industrial reliability. Scaling up fabrication using industrial molding techniques and evaluating cost–performance comparisons with conventional materials such as MDF, plywood, and glass fiber composites will further strengthen industrial relevance. Finally, extending this research toward bio-based resin systems and conducting full life-cycle assessments (LCA) will help position jute–MgO bio-nanocomposites as viable, sustainable alternatives for commercial applications.

## Data Availability

The datasets used and/or analyzed during the current study available from the corresponding author on reasonable request.

## Abbreviations

MgO: Magnesium Oxide
HLU: Hand Lay-Up
FTIR: Fourier Transform Infrared Spectroscopy
SEM: Scanning Electron Microscope
